# 人类免疫缺陷病毒感染相关弥漫大B细胞淋巴瘤诊断与治疗中国专家共识（2025年版）

**DOI:** 10.3760/cma.j.cn121090-20250703-00313

**Published:** 2025-09

**Authors:** 中华医学会血液学分会淋巴细胞疾病学组 中国抗癌协会（CACA）淋巴瘤整合康复专委会 中国临床肿瘤学会（CSCO）淋巴瘤专家委员会中西部免疫缺陷相关淋巴瘤诊治协作组（CALL）

## Abstract

人类免疫缺陷病毒感染相关弥漫大B细胞淋巴瘤（human immunodeficiency virus infection related diffuse large B cell lymphoma，HIV^+^ DLBCL）发病率低，诊断具有挑战性，治疗缺乏规范性。为加强我国临床医师对HIV^+^ DLBCL的认识，提高诊断及治疗水平，中华医学会血液学分会淋巴细胞疾病学组、中国抗癌协会（CACA）淋巴瘤整合康复专委会、中国临床肿瘤学会（CSCO）淋巴瘤专家委员会中西部免疫缺陷相关淋巴瘤诊治协作组（CALL）组织相关专家，讨论并形成本共识。

## Abstract (en)

Human immunodeficiency virus infection related diffuse large B cell lymphoma（HIV^+^ DLBCL）exhibits a low incidence and a challenging diagnosis, with a lack of standardized treatment protocols. To improve the understanding of HIV^+^ DLBCL among clinical physicians in China and enhance diagnostic and treatment levels, the Lymphoid Disease Group, Chinses Society of Hematology, Chinese Medical Association, Lymphoma Integrative Rehabilitation Professional Committee of Chinese Anti-Cancer Association（CACA）and the Central and Western China AIDS Lymphoma League（CALL）, Lymphoma Expert Committee of Chinese Society of Clinical Oncology（CSCO）have organized relevant experts to formulate this consensus.

艾滋病即获得性免疫缺陷综合征（acquired immunodeficiency syndrome，AIDS），其病原体为人类免疫缺陷病毒（human immunodeficiency virus，HIV），亦称艾滋病病毒。据联合国艾滋病规划署（The Joint United Nations Programme on HIV/AIDS，UNAIDS）统计，截至2024年底，全球现存活HIV/AIDS患者4 080万例，当年新发HIV感染者130万例，有3 160万例正在接受抗反转录病毒治疗（anti-retroviral therapy，ART）[1]。最新的生存率统计结果显示，HIV感染者预期寿命为73.9岁，健康对照组中位预期寿命为80.0岁[2]。

恶性肿瘤是HIV感染相关死亡的重要原因。2017年以来，HIV感染相关非霍奇金淋巴瘤（HIV infection related non-Hodgkin lymphoma，HIV^+^ NHL）的发生率高于卡波西肉瘤，居于艾滋病定义肿瘤的首位，发病率为（100～300）/10万（以HIV感染人群为基数）[3]。2022年世界卫生组织（World Health Organization，WHO）第五版淋巴造血组织肿瘤分类中将HIV感染相关淋巴瘤明确归为一个独立亚型：与免疫缺陷和免疫调节障碍相关的淋巴组织增生和淋巴瘤（lymphoid proliferations and lymphomas associated with immune deficiency and dysregulation），主要亚型包括弥漫大B细胞淋巴瘤（diffuse large B cell lymphoma，DLBCL）、伯基特淋巴瘤（Burkitt lymphoma，BL）、原发性中枢神经系统淋巴瘤（primary central nervous system lymphoma，PCNSL）、浆母细胞淋巴瘤（plasmablastic lymphoma，PBL）、原发性渗出性淋巴瘤（primary exudative lymphoma，PEL）和外周T细胞淋巴瘤（peripheral T-cell lymphoma，PTCL）[4]–[7]。HIV感染相关DLBCL（HIV infection related DLBCL，HIV^+^ DLBCL）是HIV^+^ NHL中最常见的病理亚型，占67.1％[8]。HIV感染者的免疫功能缺陷使HIV^+^ DLBCL与非HIV感染者具有不同的特点，抗淋巴瘤治疗需要密切关注患者的免疫缺陷状况。由于HIV^+^ DLBCL临床病例仍少见，且患者分散在感染内科、血液内科及肿瘤科，对该疾病的认识尚未统一，诊治尚不规范。为加强我国临床医师对HIV^+^ DLBCL的认知，提高诊断、鉴别诊断及治疗水平，中华医学会血液学分会淋巴细胞疾病学组、中国抗癌协会（CACA）淋巴瘤整合康复专委会、中国临床肿瘤学会（CSCO）淋巴瘤专家委员会中西部免疫缺陷相关淋巴瘤诊治协作组（CALL）组织血液病学、肿瘤学、病理学、药学、感染病学、流行病学等领域专家，基于我国临床实践，结合国内外最新研究进展，讨论并制定本共识。

一、流行病学与生存

HIV感染相关淋巴瘤中，95％以上为B细胞来源[7]。来自欧洲11个医学中心的回顾性研究报道，HIV^+^ DLBCL的中位发病年龄为47（23～83）岁[9]。我国一项多中心回顾性研究显示，中国HIV^+^ DLBCL患者的中位发病年龄同样为47（21～90）岁[10]。ART前时代，HIV^+^ DLBCL患者的中位生存期仅5～8个月[11]。ART后时代，美国SEER数据库共分析2001–2016年新诊断的8 624例DLBCL患者，其中HIV^+^ DLBCL患者2 156例，HIV^+^ DLBCL患者5年总生存（OS）率为53％[12]。国内一项多中心回顾性研究分析了2011–2021年273例HIV^+^ DLBCL患者，5年OS率为54.6％[8]。

二、生物学特征与临床表现

按细胞起源分类，HIV^+^ DLBCL中生发中心起源（GCB）更常见，占75％，而非GCB（non-GCB）仅占约25％，且MYC或BCL-6易位及P53突变更常见[13]。HIV^+^ DLBCL的临床表现也与HIV阴性患者存在一定的差异，表现出更高的侵袭性，Ki-67通常大于90％，发病年龄更低，分期更晚[14]。最常见的临床表现为浅表淋巴结肿大，可伴发热、腹痛、骨痛、鼻塞、咳嗽、胸闷、吞咽困难、纳差和乏力等临床表现。常伴随B症状、大包块（最大直径≥7.5 cm）及结外受累[14]。最常见的结外累及部位是胃肠道（15％～50％），其他常见的结外累及部位包括骨髓（13％～22％）、中枢神经系统（5％～15％）、肝和肺（均≤5％）[15]–[17]。

三、检查

HIV^+^ DLBCL的检查需在HIV阴性DLBCL的基础上额外注意病毒学和免疫功能的监测，结合病史、体格检查、实验室检查、影像学检查和病理学检查等综合考虑。

1. 病史采集：包括HIV感染史与治疗史，发热、盗汗、体重减轻等B症状，并进行体能状况评分、营养状况评分等。

2. 体格检查：应注意不同区域的淋巴结（包括浅表淋巴结、韦氏环等）是否肿大，肝脾是否肿大，伴随体征及一般情况等。

3. 实验室检查：血尿便常规、生化全套、乳酸脱氢酶（LDH）、β_2_微球蛋白（β_2_-microglobulin，β_2_-MG）、HIV RNA、免疫细胞亚群（包含CD4^+^ T淋巴细胞计数）等。同时筛查是否合并其他病原体感染，包括EB病毒（EBV）、乙型肝炎病毒（HBV）、丙型肝炎病毒（HCV）、巨细胞病毒（CMV）、结核分枝杆菌（TB）、非结核分枝杆菌（NTM）、梅毒、TORCH等；由于HIV^+^ DLBCL侵袭性高，初诊时需行骨髓穿刺（包括骨髓涂片、流式细胞术和细胞病理学检查）、腰椎穿刺（包括脑脊液常规、生化、流式细胞术和细胞病理学检查）等以确定是否侵犯骨髓与中枢神经系统；育龄期妇女需行妊娠试验。

4. 影像学检查：包括增强CT、MRI（中枢神经系统可疑受累者进行受累部位的MRI检查）、正电子发射计算机体层成像（PET-CT）、内镜检查（适用于胃肠道可疑受累等情况）、心电图检查和超声心动图（有心血管基础疾病、高龄或拟用蒽环类药物者）等。

5. 病理学检查：病理学检查是诊断HIV^+^DLBCL的金标准。对于淋巴结病灶，应尽量完整地切除淋巴结。如果病灶位于浅表部位，应优先选择颈部、锁骨上或腋窝淋巴结进行切除。只有在无法安全有效地切除病变组织的情况下，才考虑使用粗针穿刺。HIV^+^ DLBCL的病理诊断需综合组织形态学、免疫组织化学、分子遗传学及流式细胞术等，同时需结合患者的临床特征。

（1）形态学：HIV^+^ DLBCL是一类由中等大小或大肿瘤性B淋巴细胞弥漫性增生形成的肿瘤，肿瘤细胞核的大小超过或等于组织细胞细胞核，或是正常淋巴细胞的2倍以上。

（2）免疫组织化学：HIV^+^ DLBCL表达全B细胞标志物，如CD19、CD20、CD22、CD79a和PAX5；需要额外关注EBV相关抗原（如LMP1、EBNA2）及EBV编码的小RNA（EBER）的表达情况。HIV^+^ DLBCL的诊断应遵循2022版WHO分类标准，细分为GCB及non-GCB[5]。

（3）分子遗传学：完善MYC、BCL-2的染色体核型或荧光原位杂交（FISH）。同时具有MYC、BCL-2重排的病例，依据形态学特征进一步归类为DLBCL/高级别B细胞淋巴瘤伴MYC和BCL-2基因重排，亦称“双打击”淋巴瘤，提示预后不良。

（4）其他：实体组织流式细胞术、淋巴细胞抗原受体基因重排检测、二代基因测序等，是常规病理学检查的重要补充。

四、诊断、鉴别诊断、分期

1. 诊断：具有典型DLBCL的临床表现，并有明确的HIV感染病史，需同时结合患者的体格检查、实验室检查、影像学检查和病理学检查结果等进行诊断。

2. 鉴别诊断：

（1）感染性淋巴结肿大：HIV感染者易合并多种感染性疾病，如TB、NTM、CMV、马尔尼菲篮状菌感染等，上述感染均可引起淋巴结肿大，因此需要通过病史、临床表现、影像学检查和病理学检查等进行鉴别诊断。

（2）转移性肿瘤：某些实体肿瘤如肺癌、乳腺癌、胃肠道肿瘤等可发生淋巴结转移，通过病理学检查可鉴别。

3. 分期：目前尚无HIV^+^ DLBCL特有的分期体系，与普通DLBCL采用同样标准：2014年Lugano分期系统[18]，同时根据患者的全身症状分为A组（无B症状）和B组（有B症状）。

五、治疗

1. 一线治疗方案推荐：

（1）诱导治疗：与HIV阴性DLBCL首选R-CHOP（利妥昔单抗+环磷酰胺+长春新碱+阿霉素+泼尼松）方案不同，HIV^+^ DLBCL的一线治疗方案尚存在争议。一项来自法国的前瞻性队列研究显示，接受R-CHOP方案治疗的HIV^+^ DLBCL患者的2年OS率为75％[19]。国内一项回顾性研究发现，接受R-CHOP方案治疗的HIV^+^ DLBCL患者的2年OS率仅为53.8％[20]。Barta等[21]分析了来自19项前瞻性临床研究的1 546例HIV^+^ NHL患者，结果显示，对于HIV^+^ DLBCL患者，一线应用R-EPOCH方案较R-CHOP方案能更明显地改善患者的OS（*HR*＝0.72，95％*CI*：0.52～0.99，*P*＝0.046）。

结合我国的临床工作实践，本共识推荐R-DA-EPOCH方案作为初诊HIV^+^ DLBCL患者的一线诱导方案，推荐化疗6～8个周期，21 d为1个周期。R-DA-EPOCH方案初始用药剂量：利妥昔单抗375 mg/m^2^，第0天；依托泊苷50 mg/m^2^，第1～4天；阿霉素10 mg/m^2^，第1～4天；长春新碱0.4 mg/m^2^（最大剂量0.5 mg）第1～4天；环磷酰胺750 mg/m^2^（CD4^+^ T淋巴细胞计数>200个/µl）、375 mg/m^2^（CD4^+^ T淋巴细胞计数50～200个/µl）、187.5 mg/m^2^（CD4^+^ T淋巴细胞计数<50个/µl）第5天；泼尼松60 mg/m^2^，口服，第1～5天。

对于CD4^+^ T淋巴细胞计数<50个/µl的患者，一般不推荐使用利妥昔单抗。闵海燕等[22]的研究显示，应用利妥昔单抗也可以使CD4^+^ T淋巴细胞计数<50个/µl的患者获益。建议在使用利妥昔单抗的过程中严密检测，积极防治感染。

由于患者合并免疫功能异常，大剂量化疗后更易出现重度骨髓抑制，本共识推荐所有患者化疗后行长效粒细胞集落刺激因子（G-CSF）预防严重的粒细胞缺乏。对于肿瘤负荷大的患者，为预防肿瘤溶解综合征发生，大剂量化疗前可先行小剂量环磷酰胺联合地塞米松（环磷酰胺200 mg×3 d，地塞米松10 mg×3 d）治疗以降低肿瘤负荷。对于发生在消化系统的HIV^+^ DLBCL，如果出现急性肠梗阻、消化道穿孔、肠套叠、经内镜或介入不能控制的出血等情况应考虑手术治疗，术后根据患者的恢复情况及时开始全身化疗。

（2）自体造血干细胞移植巩固治疗：对于年轻中高危或高危，伴随大肿块（肿块直径≥7.5 cm）、双表达或双打击的HIV阴性DLBCL患者，一般建议诱导化疗后行自体造血干细胞移植[23]–[25]。一项前瞻性、多中心、Ⅱ期临床研究探索自体造血干细胞移植在HIV阳性淋巴瘤患者中的疗效，共纳入40例患者，其中DLBCL 16例，结果显示，HIV^+^ NHL患者的2年OS率83.5％，与普通NHL的生存无差异[26]。美国一项多中心回顾性研究显示，接受自体造血干细胞移植的HIV阳性淋巴瘤患者的住院费用、移植相关感染率和住院时间均与普通淋巴瘤类似[27]。HIV^+^ DLBCL合并HIV感染，疾病侵袭性高，进展快，易发生结外受累。因此本共识推荐，对于年龄小于70岁且体能状况较好的HIV^+^ DLBCL患者，建议在有经验的治疗中心诱导缓解后尽快行自体造血干细胞移植巩固治疗。

（3）放射治疗：化疗前大肿块（肿块直径≥ 7.5 cm）或结外器官受累、化疗后未达完全缓解（CR）是放疗适应证。局限期患者短疗程化疗后联合放疗可取得与长疗程单纯化疗相同的疗效，足量化疗后联合放疗可进一步提高疗效。化疗后CR推荐放疗剂量为30～36 Gy，化疗后部分缓解（PR）或疾病稳定（SD）推荐放疗剂量为30～40 Gy，而化疗后进展行挽救放疗时应予更高剂量40～50 Gy。

（4）维持治疗：目前关于HIV^+^ DLBCL维持治疗的数据较少，本共识不推荐维持治疗。

（5）中枢神经系统预防：鉴于HIV^+^ DLBCL患者Ki-67增殖指数明显增高、多合并TP53突变，中枢神经系统侵犯风险高[28]。因此，本共识推荐所有初诊患者均行两药或三药（阿糖胞苷+甲氨蝶呤±地塞米松）鞘内注射预防和（或）静脉输注大剂量甲氨蝶呤治疗。

2. 复发/难治方案推荐：HIV^+^ DLBCL患者复发后预后极差，优先推荐临床研究。此外，复发/难治患者推荐选择其他与R-DA-EPOCH方案无交叉耐药的二线方案（如R-DHAP、R-ICE、R-GDP、R-ESHAP、R-GemOx、R-MINE、Pola-BR、R2、BR方案等）化疗，缓解后如一线未行自体造血干细胞移植，则尽快行自体造血干细胞移植。对于≥2次复发/进展患者，则推荐异基因造血干细胞移植、CAR-T细胞或新药治疗。

3. 新药探索：目前关于HIV^+^ DLBCL的临床研究尚少，其中基因治疗、布鲁顿酪氨酸激酶抑制剂（BTKi）、组蛋白去乙酰化酶抑制剂（HDACi）、CAR-T细胞治疗等正在临床研究阶段（表1）。国内一项前瞻性、单臂、开放标签临床研究评估塞利尼索联合R-EPOCH（XR-EPOCH）方案治疗初诊HIV^+^ DLBCL患者的疗效和安全性（ChiCTR2300069941），共入组10例患者，均获得治疗反应，其中8例获得CR，中位随访23（11～26）个月，2年OS率为85.7％，且安全性可控，初步显示加入新药可进一步提高患者疗效[28]。另外，免疫调节剂联合PD1/PD-L1抗体[29]、HDACi联合标准化疗[30]、异基因造血干细胞移植[31]、CD20/CD3双特异性抗体、CD19单克隆抗体、BCL-2抑制剂等在HIV^+^ DLBCL治疗中的应用亦在探索（表2）。本共识呼吁，未来的临床研究不应除外所有合并HIV感染的患者，HIV RNA转阴、CD4^+^ T淋巴细胞计数>200个/µl的非活动期HIV感染者应纳入临床研究。HIV^+^ DLBCL的治疗路径参照图1。

**表1:** 目前正在开展的关于HIV^+^ DLBCL的临床研究

治疗方案	开始时间	注册号	研究阶段	研究人群
基因治疗联合化疗	2015年	NCT02337985	未入组	HIV^+^ BL、HIV^+^ DLBCL、HIV^+^ PBL
伊布替尼联合R-EPOCH方案	2018年	NCT03220022	招募中	Ⅱ～Ⅳ期HIV^+^ DLBCL
嵌合抗原受体T细胞	2024年	NCT05077527	未入组	HIV^+^ DLBCL
伏立诺他联合R化疗	2010年	NCT01193842	结束	HIV^+^ PBL、HIV^+^ PEL、HIV^+^ DLBCL

**注** HIV：人类免疫缺陷病毒；DLBCL：弥漫大B细胞淋巴瘤；BL：伯基特淋巴瘤；PBL：浆母细胞淋巴瘤；PEL：原发性渗出性淋巴瘤；R-EPOCH：利妥昔单抗+依托泊苷+环磷酰胺+阿霉素+长春新碱+泼尼松；R：利妥昔单抗

**表2:** PD1/PD-L1抗体、HDACi、异基因造血干细胞移植等治疗HIV^+^ DLBCL的疗效和安全性

治疗方案	例数	CR率	生存情况	安全性
帕博利珠单抗+泊马度胺[29]	4	25％	3例因PD死亡	1例患者发生2级甲状腺功能减退
伏立诺他+R-EPOCH方案[30]	30	70％	3年OS率70％	4例患者因AE停止治疗
异基因造血干细胞移植[31]	17（其中NHL 3例）	76.5％	2年OS率52.3％	100 d 3～4级GVHD发生率11.8％，1年慢性GVHD发生率17.6％

**注** HDACi：组蛋白去乙酰化酶抑制剂；HIV：人类免疫缺陷病毒；DLBCL：弥漫大B细胞淋巴瘤；R-EPOCH：利妥昔单抗+依托泊苷+环磷酰胺+阿霉素+长春新碱+泼尼松；NHL：非霍奇金淋巴瘤；CR：完全缓解；PD：疾病进展；OS：总生存；AE：不良反应；GVHD：移植物抗宿主病

**图1:**
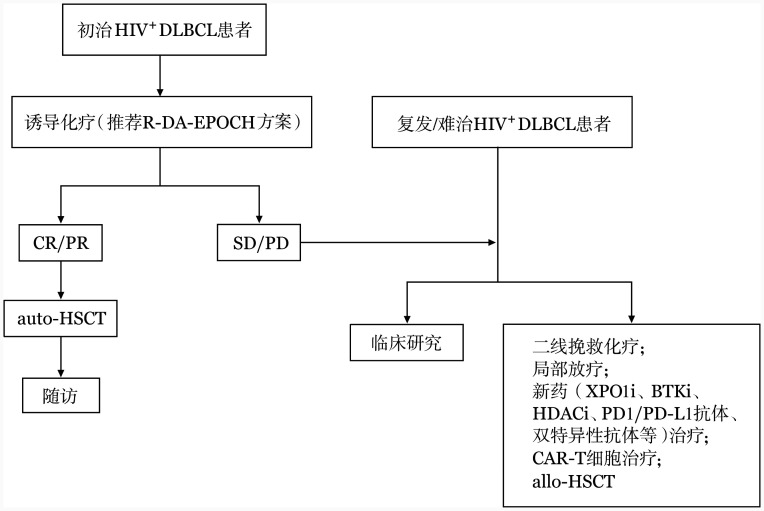
人类免疫缺陷病毒感染相关弥漫大B细胞淋巴瘤（HIV^+^ DLBCL）的治疗路径 **注** R-DA-EPOCH：利妥昔单抗+依托泊苷+环磷酰胺+阿霉素+长春新碱+泼尼松；CR：完全缓解；PR：部分缓解；auto-HSCT：自体造血干细胞移植；SD：疾病稳定；PD：疾病进展；XPO1i：核输出蛋白1抑制剂；BTKi：布鲁顿酪氨酸激酶抑制剂；HDACi：组蛋白去乙酰化酶抑制剂；PD1：程序性死亡受体1；PD-L1：程序性死亡配体1；CAR-T细胞：嵌合抗原受体T细胞；allo-HSCT：异基因造血干细胞移植

4. 抗HIV治疗方案：HIV应按《中华人民共和国传染病防治法》乙类传染病管理。对以淋巴瘤为首发症状的HIV患者，在保护患者隐私的同时，应在诊断24 h内上报。参照《中国艾滋病诊疗指南（2024版）》[32]，一旦确诊HIV感染，无论CD4^+^ T淋巴细胞水平如何，均建议尽快开始ART。若患者存在严重的机会性感染或处于慢性疾病急性发作期，应先处理机会性感染，病情稳定后尽早开始ART。对于新诊断的HIV^+^ DLBCL患者，尤其是高肿瘤负荷、伴B症状者，本共识建议在积极有效抗肿瘤治疗的同时或1周内启动ART，避免发生肿瘤免疫重建后快速进展，失去治疗机会。启动ART后需终身治疗且尽量不要因抗肿瘤治疗而中断。

推荐应用含整合酶抑制剂的方案，对于HIV RNA≥1.0×10^5^拷贝/ml的高病毒载量患者或存在消化功能障碍患者，在化疗过程中可联合使用静脉剂型ART药物融合抑制剂如艾博卫泰（ABT），以快速抑制病毒复制，且与抗肿瘤治疗药物的相互作用风险低。尽量规避应用齐多夫定等容易引起骨髓抑制的药物及与DLBCL治疗方案相互作用的药物。具体抗病毒方案参照《中国艾滋病诊疗指南（2024版）》[32]，建议感染科医师和血液肿瘤科医师会诊制定抗病毒治疗方案。

5. 感染的预防：HIV^+^ DLBCL患者一旦出现感染，病情进展迅速，快速获得病原学结果困难，因此，靶向化疗期间的预防性抗微生物治疗十分必要。预防性抗感染策略常需覆盖细菌、耶氏肺孢子菌、带状疱疹病毒。化疗期间均建议使用阿昔洛韦或伐昔洛韦预防带状疱疹病毒，即使CD4^+^ T淋巴细胞计数较高（>200个/µl）时同样建议预防性应用，因为化疗联合甲泼尼龙通常会导致CD4^+^ T淋巴细胞计数下降。所有患者均需要使用复方磺胺甲噁唑预防耶氏肺孢子菌肺炎；建议接受强化疗的患者预防性应用氟喹诺酮类药物治疗，因为这类患者可能会出现长期（>7 d）中性粒细胞减少症和黏膜炎。通常不建议对真菌进行常规一级预防；然而，对于存在严重免疫抑制（如CD4^+^ T淋巴细胞计数<100个/µl）、预期长期中性粒细胞减少、接受高剂量甲氨蝶呤或阿糖胞苷方案（易引起黏膜炎）的患者，可以考虑预防性应用氟康唑抗真菌治疗。如果CD4^+^ T淋巴细胞计数<100个/µl，建议动态监测CMV变化，必要时予更昔洛韦、缬更昔洛韦或膦甲酸钠治疗。具体可参照《中国艾滋病诊疗指南（2024版）》[32]和《造血干细胞移植治疗淋巴瘤中国专家共识（2018版）》[33]。

HIV^+^ DLBCL总体发病率低，大多数医院治疗经验有限，建议进行多学科诊疗（MDT），需特别注意请感染科会诊指导抗HIV治疗和机会性感染的防治。

六、疗效评估

与普通DLBCL患者一样，采用2014 Lugano淋巴瘤治疗效果评价标准[18]评价疗效，治疗效果分为基于CT和（或）MRI评价的影像学缓解和基于PET-CT评价的代谢缓解，PET-CT评价代谢缓解的依据是Deauville标准的PET 5分法（five-point scale，5PS）。疗效评估分为CR、PR、SD和疾病进展。

七、随访

进行全生命周期随访，即完成治疗后的前2年应每3个月进行1次随访，包括病史、查体、血细胞计数、生化常规、免疫细胞亚群、HIV RNA及影像学检查。完成治疗后第2～5年每6个月进行1次随访。5年后每年复查1次，终身随访。当临床出现可疑复发征象时应立即检查，对于新出现的病灶应尽量进行活检，以明确病理诊断。随访内容除监测复发外，需特别关注第二肿瘤的发生、年轻患者后续的生育情况及生活质量。

八、预后

随着ART和免疫化疗的联合应用，HIV^+^ DLBCL的整体预后已经不亚于HIV阴性DLBCL[14]。HIV^+^ DLBCL患者的预后主要与其淋巴瘤临床分期和HIV感染后病毒载量以及免疫功能状态相关。临床分期早期及HIV RNA持续低水平患者的预后常相对较好，诊断时表现为中枢神经系统受累及巨大包块的患者预后差。国内开发出一种针对HIV^+^ DLBCL患者的预后模型（AIDS related DLBCL prognostic index，ARDPI）用于新诊断HIV^+^ DLBCL患者的预后评估及治疗指导等，具体包括以下7个因素：年龄、淋巴细胞/单核细胞比例、淋巴瘤细胞CD5表达情况、血液EBV-DNA拷贝数、CD4/CD8比例、中枢神经系统受累和AIDS ART。根据以上7个因素在可视化列线图上的总得分，可将新诊断HIV^+^ DLBCL患者分为低危、中危及高危，并能预测其生存率，其预测精度优于国际预后指数（IPI）评分和美国国立综合癌症网络（NCCN）-IPI评分，临床上可以根据ARDPI预后分层对患者实施个体化精准治疗，如对高危患者采用更强的化疗方案等，有望改善HIV^+^ DLBCL患者的整体预后[34]。

九、总结

HIV^+^ DLBCL总体发病率较低，中国幅员辽阔，各地区发展不均衡，导致诊疗水平参差不齐。近年来，HIV^+^ DLBCL规范化诊疗在我国专业领域学者的努力下已有长足进步，如规范ART联合免疫靶向治疗。另外，一些新的治疗方案如加入新药（XPO1抑制剂、PD1抑制剂等）、自体造血干细胞移植的广泛应用也取得了相应的临床疗效。本文为HIV^+^ DLBCL提供治疗方案推荐，在新药应用方面力求为临床医师提供参考，进一步推动HIV^+^ DLBCL规范化诊疗发展，提高患者的生存率及生活质量。

## References

[1] UNAIDS global AIDS update 2025. AIDS, crisis and the power to transform[EB/OL].

[2] Lohse N, Obel N (2016). Update of survival for persons with HIV infection in Denmark[J]. Ann Intern Med.

[3] Yarchoan R, Uldrick TS (2018). HIV-associated cancers and related diseases[J]. N Engl J Med.

[4] (1992). 1993 revised classification system for HIV infection and expanded surveillance case definition for AIDS among adolescents and adults[J]. MMWR Recomm Rep.

[5] Alaggio R, Amador C, Anagnostopoulos I (2022). The 5th edition of the World Health Organization Classification of Haematolymphoid Tumours: Lymphoid Neoplasms[J]. Leukemia.

[6] Vaccher E, Chadburn A, Gloghini A (2023). Lymphomas included in the AIDS case definition: an update 30 years later[J]. Lancet HIV.

[7] Carbone A, Vaccher E, Gloghini A (2022). Hematologic cancers in individuals infected by HIV[J]. Blood.

[8] Wang C, Wu Y, Li S (2025). A national, multicenter, retrospective study of human immunodeficiency virus infection-associated lymphoma in China[J]. Clin Exp Med.

[9] Pagani C, Rusconi C, Dalla Pria A (2024). MYC rearrangements in HIV-associated large B-cell lymphomas: EUROMYC, a European retrospective study[J]. Blood Adv.

[10] Wang C, Wu Y, Liu J (2023). Impact of initial chemotherapy cycles and clinical characteristics on outcomes for HIV-associated diffuse large B cell lymphoma patients: The Central and Western China AIDS Lymphoma League 001 study (CALL-001 study)[J]. Front Immunol.

[11] Kaplan LD, Straus DJ, Testa MA (1997). Low-dose compared with standard-dose m-BACOD chemotherapy for non-Hodgkin's lymphoma associated with human immunodeficiency virus infection. National Institute of Allergy and Infectious Diseases AIDS Clinical Trials Group[J]. N Engl J Med.

[12] Valcarcel B, Schonfeld SJ, Shiels MS (2025). Survival outcomes in diffuse large B-cell lymphoma patients with and without HIV in the United States from 2001 to 2016: a population-based analysis[J]. Haematologica.

[13] McGee-Avila JK, Suneja G, Engels EA (2024). Cancer Treatment disparities in people with HIV in the United States, 2001-2019[J]. J Clin Oncol.

[14] 王 超雨, 刘 俊, 梁 喜平 (2022). 63例HIV相关弥漫大B细胞淋巴瘤临床特征及预后分析:国内单中心真实世界研究[J]. 中华血液学杂志.

[15] Imrie KR, Sawka CA, Kutas G (1995). HIV-associated lymphoma of the gastrointestinal tract: the University of Toronto AIDS-Lymphoma Study Group experience[J]. Leuk Lymphoma.

[16] Beck PL, Gill MJ, Sutherland LR (1996). HIV-associated non-Hodgkin's lymphoma of the gastrointestinal tract[J]. Am J Gastroenterol.

[17] Diamond C, Taylor TH, Aboumrad T (2006). Changes in acquired immunodeficiency syndrome-related non-Hodgkin lymphoma in the era of highly active antiretroviral therapy: incidence, presentation, treatment, and survival[J]. Cancer.

[18] Cheson BD, Fisher RI, Barrington SF (2014). Recommendations for initial evaluation, staging, and response assessment of Hodgkin and non-Hodgkin lymphoma: the Lugano classification[J]. J Clin Oncol.

[19] Besson C, Lancar R, Prevot S (2017). Outcomes for HIV-associated diffuse large B-cell lymphoma in the modern combined antiretroviral therapy era[J]. AIDS.

[20] Wang C, Liu J, Lei H (2022). Clinical characteristics and outcomes of newly diagnosed patients with HIV-associated aggressive B-cell NHL in China[J]. J Cell Mol Med.

[21] Barta SK, Xue X, Wang D (2013). Treatment factors affecting outcomes in HIV-associated non-Hodgkin lymphomas: a pooled analysis of 1546 patients[J]. Blood.

[22] 闵 海燕, 李 侠, 汪 习成 (2019). 艾滋病相关恶性淋巴瘤83例临床治疗体会[J]. 重庆医学.

[23] Stiff PJ, Unger JM, Cook JR (2013). Autologous transplantation as consolidation for aggressive non-Hodgkin's lymphoma[J]. N Engl J Med.

[24] Chiappella A, Martelli M, Angelucci E (2017). Rituximab-dose-dense chemotherapy with or without high-dose chemotherapy plus autologous stem-cell transplantation in high-risk diffuse large B-cell lymphoma (DLCL04): final results of a multicentre, open-label, randomised, controlled, phase 3 study[J]. Lancet Oncol.

[25] Wen Q, Gao L, Xiong JK (2021). High-dose chemotherapy combined with autologous hematopoietic stem cell transplantation as frontline therapy for intermediate/high-risk diffuse large B cell lymphoma[J]. Curr Med Sci.

[26] Alvarnas JC, Le Rademacher J, Wang Y (2016). Autologous hematopoietic cell transplantation for HIV-related lymphoma: results of the BMT CTN 0803/AMC 071 trial[J]. Blood.

[27] Ruiz M, Rubens M, Ramamoorthy V (2023). Comparison of inpatient outcomes between HIV positive and negative hospitalizations for autologous stem cell transplant treatment among lymphoid malignancies[J]. Clin Lymphoma Myeloma Leuk.

[28] Wang C, Zhou Y, Zeng C (2023). Selinexor in combination with R-EPOCH for patients with previously untreated HIV-associated diffuse large B cell lymphoma (DLBCL)[J]. Blood.

[29] Lurain K, Ramaswami R, Mangusan R (2021). Use of pembrolizumab with or without pomalidomide in HIV-associated non-Hodgkin's lymphoma[J]. J Immunother Cancer.

[30] Ramos JC, Sparano JA, Chadburn A (2020). Impact of Myc in HIV-associated non-Hodgkin lymphomas treated with EPOCH and outcomes with vorinostat (AMC-075 trial)[J]. Blood.

[31] Ambinder RF, Wu J, Logan B (2019). Allogeneic hematopoietic cell transplant for HIV patients with hematologic malignancies: the BMT CTN-0903/AMC-080 trial[J]. Biol Blood Marrow Transplant.

[32] 中华医学会感染病学分会艾滋病学组, 中国疾病预防控制中心 (2024). 中国艾滋病诊疗指南(2024版)[J]. 中华传染病杂志.

[33] 中国抗癌协会血液肿瘤专业委员会, 中华医学会血液学分会白血病淋巴瘤学组, 中国临床肿瘤学会抗淋巴瘤联盟 (2018). 造血干细胞移植治疗淋巴瘤中国专家共识(2018版)[J]. 中华肿瘤杂志.

[34] Yang T, Lei H, Li J (2025). A visual nomogram survival prediction model in acquired immune deficiency syndrome (AIDS)-related diffuse large B-cell lymphoma[J]. J Med Virol.

